# High molecular weight fibroblast growth factor 2 induces apoptosis by interacting with complement component 1 Q subcomponent–binding protein in vitro

**DOI:** 10.1002/jcb.27131

**Published:** 2018-08-29

**Authors:** Xiaobing Hong, Zelin Yu, Zhonglin Chen, Hongyan Jiang, Yongdong Niu, Zhanqin Huang

**Affiliations:** ^1^ The Second Affiliated Hospital, Shantou University Medical College Shantou China; ^2^ Department of Pharmacology Shantou University Medical College Shantou China

**Keywords:** apoptosis, complement component 1 Q subcomponent–binding protein, coimmunoprecipitation, high molecular weight FGF2, mitochondrion

## Abstract

Fibroblast growth factor 2 (FGF2) is a multifunctional cell growth factor that regulates cell proliferation, differentiation, adhesion, migration, and apoptosis. FGF2 has multiple isoforms, including an 18‐kDa low molecular weight isoform (lo‐FGF2) and 22‐, 23‐, 24‐, and 34‐kDa high molecular weight isoforms (hi‐FGF2). Hi‐FGF2 overexpression induces chromatin compaction, which requires the mitochondria and leads to apoptosis. Complement component 1 Q subcomponent–binding protein (C1QBP) plays an important role in mitochondria‐dependent apoptosis by regulating the opening of the mitochondrial permeability transition pore. However, the interaction between C1QBP and hi‐FGF2 and its role in hi‐FGF2–mediated apoptosis remain unclear. Here, we found that hi‐FGF2 overexpression induced depolarization of the mitochondrial membrane, cytochrome *c* release into the cytosol, and a considerable increase in *C1QBP* messenger RNA and protein expression. Furthermore, coimmunoprecipitation results showed that the mitochondrial protein, C1QBP, interacts with hi‐FGF2. *C1QBP* knockdown using small interfering RNA significantly decreased the localization of hi‐FGF2 to the mitochondria and increased the rate of apoptosis. Our results highlight a novel mechanism underlying hi‐FGF2–induced, mitochondria‐driven cell death involving the direct interaction between hi‐FGF2 and C1QBP and the upregulation of C1QBP expression.

## 1 INTRODUCTION

Fibroblast growth factor 2 (FGF2) is expressed as an AUG‐initiated 18‐kDa isoform (lo‐FGF2) or CUG‐initiated 21 to 34 kDa isoforms (hi‐FGF2), whose levels vary depending on cell and tissue type and developmental stage.^1^ In cell cultures, recombinant FGF2 isoforms have been overexpressed intracellularly to examine their roles in cellular responses, such as proliferation, differentiation, and migration, and in maintaining cell viability. The differential subcellular localization and trafficking of FGF2 isoforms are indications that they possess distinct functions. A number of reports have suggested that intracrine hi‐FGF2 signaling may play a role in inhibiting cell proliferation and promoting cell death,^2^, ^3^ whereas lo‐FGF2 is released from cells and signals via its interaction with high‐affinity transmembrane FGF receptors (FGFR1‐4) in a paracrine or autocrine manner,^2^, ^3^ promoting cell proliferation and migration.^4^, ^5^, ^6^

Several studies have reported an association between cell death and hi‐FGF2 overexpression in stable cell lines. Hi‐FGF2 accumulates in human skin fibroblasts under heat or oxidative stress,^7^ inducing apoptosis. Overexpression of hi‐FGF2, but not lo‐FGF2, significantly increases binucleation and the formation of compacted chromatin clumps, a unique nuclear phenotype, in an intracrine manner.^8^, ^9^ Hi‐FGF2–associated chromatin compaction in cardiomyocytes appears to reflect the direct effects of hi‐FGF2 on chromatin structure that does not require mitosis.^10^ Moreover, the isoproterenol‐induced increase in the transient expression of endogenous hi‐FGF2 and the induction of cell death suggest that increased levels of hi‐FGF2 exert cytotoxic effects.^11^ The intracrine activity of hi‐FGF2 induces mitotic arrest, chromatin disruption, and cell loss and promotes the appearance of a DNA ladder, presumably due to apoptosis.^12^ A complementary DNA (cDNA) microarray–based study^13^ demonstrated that hi‐FGF2 expression in NIH3T3 fibroblasts upregulated the expression of genes associated with cell‐cycle arrest, such as *NFLX* and *NUPR1*, and tumor suppression, such as *ST5*. Hi‐FGF2–induced chromatin compaction and cell death depend on the nuclear localization of intact hi‐FGF2 and require mitochondrial involvement through an extracellular signal–regulated kinases 1 and 2(ERK1/2)–dependent pathway.^14^

Although several studies have investigated hi‐FGF2–induced apoptosis, the mechanisms underlying this process remain unclear. In the current study, we identified hi‐FGF2–interacting proteins to examine hi‐FGF2–specific effects on chromatin and apoptosis. Complement component 1 Q subcomponent–binding protein (C1QBP) localizes to different subcellular sites, predominantly the mitochondria, and is involved in RNA splicing^15^ and oxidative phosphorylation.^16^ Recent studies have shown that C1QBP regulates the opening of the mitochondrial permeability transition (MPT) pore and thus mitochondria‐driven cell death.^17^, ^18^ Because hi‐FGF2–induced chromatin compaction and cell death require mitochondria, we hypothesized that hi‐FGF2‐C1QBP interactions contribute to the hi‐FGF2–induced apoptotic phenotype. Therefore, we explored the effect of hi‐FGF2 on apoptosis and investigated whether hi‐FGF2 overexpression induces apoptosis by upregulating the expression of and binding to C1QBP through a mitochondria‐dependent pathway.

## 2 MATERIALS AND METHODS

### 2.1 Cells

Human embryonic kidney 293 (HEK293) cells were purchased from the Cell Bank of Type Culture Collection of the Chinese Academy of Sciences (Shanghai, China) and grown in Dulbecco modified Eagle medium (DMEM; Gibco, Carlsbad, CA) supplemented with 10% fetal bovine serum (Gibco) and 100 U/mL penicillin and streptomycin at 37°C in a humidified atmosphere of 5% CO_2_.

### 2.2 Plasmids

Construction and characterization of cDNA encoding DsRed‐labeled rat hi‐FGF2 were performed as described previously.^8^, ^19^

### 2.3 Transient gene transfer

Transient gene transfer was performed using the Lipofectamine 2000 Transfection Reagent (Invitrogen, Waltham, MA) according to the manufacturer's instructions. Briefly, 10 µL of Lipofectamine 2000 Transfection Reagent was mixed with 200 µL of serum‐free DMEM and incubated for 5 minutes at room temperature. DNA samples (4 μg per 35‐mm well) were diluted in 200 µL of serum‐free DMEM, mixed with diluted Lipofectamine 2000 Transfection Reagent, and incubated for 15 to 20 minutes at room temperature. Finally, this mixture was added dropwise to cells (4 × 10^5^) seeded in 35‐mm wells. The cells were incubated with the transfection mixture for 6 hours, after which the medium was refreshed. Transfection efficiency was determined after 24 to 48 hours of transfection by immunofluorescence analysis and was found to be consistently high.

### 2.4 Western blot analysis

Following transfection, cells were harvested and lysed in cold lysis buffer (50 mM of Tris‐Cl [pH 8], 3 mM of EDTA, 100 mM of NaCl, 1% Triton X‐100, 10% glycerol, 0.5 mM of PMSF, and protease inhibitor). Cell lysates were centrifuged at 12 000*g* and 4°C for 15 minutes, after which protein concentrations were determined using the BCA Protein Assay Kit (Thermo Fisher Scientific, Waltham, MA). Protein samples (50 μg) were resolved by sodium dodecyl sulfate‐polyacrylamide gel electrophoresis (SDS‐PAGE) on 12% gels and then transferred to poly(vinylidene difluoride) membranes. Protein expression was analyzed using the following commercially available antibodies: rabbit polyclonal anti‐FGF2 (1:5000; Epitomics, Cambridge, UK), monoclonal anti‐RFP (1:5000; MBL, Kyoto, Japan), rabbit polyclonal anti‐C1QBP (1:7000; Epitomics), rabbit polyclonal anti‐voltage–dependent anion channel (VDAC; 1:5000; BosterBio, Wuhan, China), mouse monoclonal anti‐FGF2 (1:5000; Abcam, Cambridge, UK), mouse monoclonal anti–cytochrome *c* (1:1000; Cell Signaling, Danvers, MA), and mouse monoclonal anti–β‐actin (1:6000; ZSGB‐Bio, Beijing, China). Next, the membranes were washed with tris‐buffered saline and Tween 20 (TBST), incubated with horseradish peroxidase–conjugated anti‐rabbit or anti‐mouse secondary antibody in 1% skim milk and TBST, and then visualized using an enhanced chemiluminescence reagent (SuperSignal Chemiluminescent Substrate; Pierce, Waltham, MA). Protein bands were analyzed using Gel‐Pro Image Analysis Software (Media Cybernetics, Rockville, MD).

### 2.5 Coimmunoprecipitation

Coimmunoprecipitation (CoIP) was performed using a CoIP Kit (Thermo Fisher Scientific) according to the manufacturer's instructions. The cells were grown to approximately 90% confluence and washed with 1× phosphate‐buffered saline. Next, 1 mL of IP buffer (20 mM of Tris‐HCl [pH 7.5], 137 mM of NaCl, 2 mM of EDTA, 25 mM of β‐glycerophosphate, 1 mM of sodium orthovanadate, 1% Triton X‐100, 1% deoxycholate, and complete protease inhibitors) was added to the cells (culture plate size, 100 × 60 mm^2^). Cell lysates were collected, incubated on ice for 5 minutes with periodic mixing, and then centrifuged at 13 000*g* and 4°C for 10 minutes. The supernatant (50 µL) was collected and IP analysis was performed by incubating the supernatant overnight on ice with the rabbit polyclonal anti‐FGF2 antibody or the anti‐RFP and rabbit polyclonal anti‐C1QBP antibodies at a concentration of 5 µg/mg lysate. Control IP experiments were performed using protein A/G agarose. Protein A/G agarose was added to a Pierce spin column and centrifuged at 1000*g* for 1 minutes. The flow‐through was discarded, and the column was washed twice with 100 µL of IP lysis/wash buffer. Antibody‐lysate samples were added to protein A/G agarose in the spin column and incubated for 1 hour. The gel slurry was washed 3 times with 200 µL of IP lysis/wash buffer and once with 100 µL of 1× conditioning buffer. Next, 50 µL of 2× lane marker nonreducing sample buffer was added to the samples and then incubated at 100°C for 5 to 10 minutes. Proteins in the samples were resolved by SDS‐PAGE and detected by Western blot analysis. As input control, 0.25% cell lysate was loaded in each lane.

### 2.6 Preparation of mitochondrial and cytosolic fractions

Mitochondrial and cytosolic fractions were prepared by differential centrifugation using the Cell Mitochondria Isolation Kit (Beyotime Institute of Biotechnology, Jiangsu, China) according to the manufacturer's instructions. Briefly, the cells were incubated on ice in 100 µL of ice‐cold mitochondrial lysis buffer for 10 minutes. Next, the cell suspension was added to a glass homogenizer and homogenized with 30 strokes using a tight pestle on ice. The homogenate obtained was centrifuged at 600*g* and 4°C for 10 minutes to remove nuclei and unbroken cells. The supernatant was collected and centrifuged again at 12 000*g* and 4°C for 30 minutes to obtain cytosolic (supernatant) and mitochondrial (pellet) fractions. Cytosolic and mitochondrial fractions were dissolved in lysis buffer and analyzed for C1QBP, FGF2, and cytochrome *c* expressions by Western blot analysis.

### 2.7 RNA interference experiments

Small interfering RNA (siRNA) oligonucleotides targeting *C1QBP* messenger RNA and control scrambled oligonucleotides (si‐Scr) were synthesized commercially (Biotend, Shanghai, China). We used 2 *C1QBP*‐targeting sequences, namely, 5′‐CUGAAUGGAAGGAUACUAA‐dTdT‐3′ (si‐*C1QBP*) and 5′‐UUAGUAUCCUUCCAUUCAG‐TdTdT‐3′ (si‐*C1QBP*‐2). Cells (4 × 10^5^) cultured in 35‐mm dishes were transfected with siRNA oligonucleotides (80 pmol) by using Lipofectamine 2000 according to the manufacturer's instructions.

### 2.8 PCR analysis

Total RNA was isolated from HEK293 cells using TRIzol reagent (TaKaRa, Dalian, China) according to the manufacturer's instructions. RNA (500 ng) was reverse‐transcribed using the PrimeScript RT Reagent Kit with gDNA Eraser (TaKaRa) to generate first‐strand cDNA. Next, the cDNA samples were analyzed by polymerase chain reaction (PCR) using the following specific primers: *FGF2* forward, 5′‐AGAAGAGCGACCCTCACATCA‐3′ and reverse, 5′‐CGGTTAGCACACACTCCTTTG‐3′ *C1QBP* forward, 5′‐CACACCGACGGAGACAAAG‐3′ and reverse, 5′‐GGGAGGGTTTTATGCTTCTGAAT‐3′ and *GAPDH* forward, 5′‐CTGGGCTACACTGAGCACC‐3′ and reverse, 5′‐AAGTGGTCGTTGAGGGCAATG‐3′. Quantitative PCR was performed with 1 µL of cDNA (1:10) and 10 µL of SYBR Premix Ex Taq II (Tli RNaseH Plus; TaKaRa) using the Illumina‐Eco Real‐Time PCR Detection System (Illumina, San Diego, CA) as described above. All PCR assays were performed in triplicate. PCR conditions were as follows: 50°C for 2 minutes, 95°C for 30 s, and 40 cycles of 95°C for 5 s and 60°C for 30 s. The housekeeping gene *GAPDH* was used for normalizing the expression levels of target genes and for monitoring assay reproducibility. Reaction mixtures lacking the cDNA template were used as negative control.

Threshold cycle (*C*_t_) numbers were determined using the Illumina‐Eco Real‐Time PCR Detection System and then transformed by the comparative *C*_t_ (ΔΔ*C*_t_) method. Target gene levels, which were normalized to those of the endogenous reference gene and were relative to calibrator levels, were determined by the ΔΔ*C*_t_ method.

### 2.9 Analysis of mitochondrial membrane potential

Cells were collected and washed twice with the culture medium. Equal amounts of cells (1 × 10^6^ cells/mL) were incubated in a culture medium containing 1× JC‐1 dye (Cayman, Ann Arbor, MI) for 15 minutes at 37°C in a 5% CO_2_ incubator according to the manufacturer's instructions. Next, the cells were washed 3 times with the medium and analyzed by flow cytometry in FL‐1 and FL‐2 channels. MPT was assessed by determining decreases in red fluorescence signal (FL‐2), indicating the presence of mitochondria with low membrane potential (Δψ_m_).

### 2.10 Data collection and statistical analysis

Nuclear compaction index (NCI) was determined by fluorescence microscopy and scoring of visual fields over the total number of hi‐FGF2–overexpressing cells per visual field. NCI represents the proportion of hi‐FGF2–overexpressing cells displaying characteristic nuclear morphology (compacted and/or fragmented chromatin). A total of 24 fields (approximately 1200 cells in total; from 3 separate coverslips/group) were scored using a low‐magnification (10×) lens. NCI or “relative NCI” (arbitrarily defining control group values as 1.0). between two groups was statistically compared using a paired Student *t* test (GraphPad InStat v5.0; GraphPad Software, La Jolla, CA).^14^ Data are presented as the mean ± standard error of the mean (SEM). Differences between groups were determined by 1‐way analysis of variance, followed by the Student‐Newman‐Keuls test using SPSS software (v17.0; SPSS Inc, Chicago, Il). *P* values < .05 were considered statistically significant.

## 3 RESULTS

### 3.1 Hi‐FGF2 overexpression depolarizes the mitochondrial membrane and releases cytochrome *c* into the cytosol

**Figure 1:**
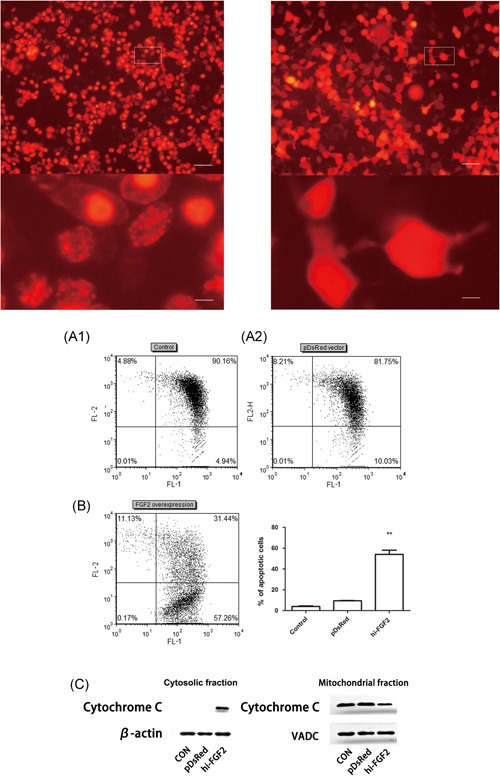
Hi‐FGF2 overexpression induces depolarization of the mitochondrial membrane and release of cytochrome *c* into the cytosol. (A) Hi‐FGF2‐pDsRed1‐N1–transfected and empty pDsRed1‐N1–transfected cells; magnification, ×100; (A1) Hi‐FGF2‐pDsRed1‐N1–transfected cells; (A2) empty pDsRed1‐N1–transfected cells. The region marked with a rectangle is enlarged and shown in the lower panel. Scale bar: 50 µm (upper panel) and 20 µm (lower panel). (B) Mitochondrial membrane potential (Δψ_m_) in hi‐FGF2–overexpressing cells. In nonapoptotic cells (high Δψ_m_), JC‐1 exists as a monomer in the cytosol (green; FL‐1) and accumulates in the mitochondria (red; FL‐2). In apoptotic cells (low Δψ_m_), JC‐1 exists in the monomeric form and stains the cytosol green. The number of cells with preserved Δψ_m_ is high in both FL‐1 and FL‐2 (top right), whereas the number of cells showing Δψ_m_ loss is high in the FL‐1 channel and low in the FL‐2 channel (bottom). Flow cytometry data are presented as the percentage of total events. Data are expressed as the mean ± SEM, n = 3; **P < .01 vs control cells. C, Cytochrome *c* levels in mitochondrial and cytosolic fractions of hi‐FGF2–overexpressing cells. β‐actin levels were used for normalization. VDAC (mitochondrial marker) levels were used to confirm the purity of the mitochondrial fractions. CON, control; SEM, standard error of the mean; VDAC, voltage dependent anion channel

HEK293 cells successfully expressed hi‐FGF2 48 hours after plasmid transfection, and transfection efficiency was approximately 80%. DsRed hi‐FGF2 fluorescence signals localized to the nucleus and were associated with a distinct nuclear phenotype characterized by multiple condensed chromatin clumps (Figure 1A1). The DsRed fluorescence signal of empty pDsRed‐transfected cells showed a diffuse cytosolic localization pattern and did not affect the cell phenotype (Figure 1A2). Figure S1 shows a quantitative assessment of the NCI, representing the proportion of overexpressing hi‐FGF2 cells with a compacted chromatin phenotype compared with cells transfected with the empty pDsRed vector. Typically, 50% to 60% of transfected cells exhibited compacted chromatin after 48 hours.

Nuclear hi‐FGF2 induces mitochondria‐mediated activation of a proapoptotic pathway. We examined the hi‐FGF2 overexpression‐induced changes in Δψ_m_ and found that hi‐FGF2 overexpression significantly increased the mitochondrial depolarization rate (57.26%; *P* < .01; Figure 1B) compared with that of control cells and empty pDsRed (red fluorescence protein vector)–transfected cells (10.03%; Figure 1B).

Next, we measured the release of cytochrome *c* from the inner mitochondrial space by Western blot analysis. Cytochrome *c* release from the mitochondria is a well‐known mechanism of triggering downstream activation of caspase‐9, which in turn cleaves caspase‐3, resulting in cell death. Results of Western blot and densitometry analyses showed a statistically significant decrease in relative mitochondrial cytochrome *c* levels in hi‐FGF2–overexpressing cells compared with that of control and pDsRed‐transfected cells (*P* < .05). Moreover, cytochrome *c* was shown to be released into the cytosol as it was present in the cytosolic fraction (Figure 1C).

### 3.2 Hi‐FGF2 overexpression upregulates C1QBP expression

C1QBP expression is upregulated under stress conditions, such as in cryptorchid rats during spermatogenic arrest, during cisplatin‐induced apoptosis in HeLa cells, or in hypoxic and nutrient‐deprived tumors.^20^, ^21^

**Figure 2:**
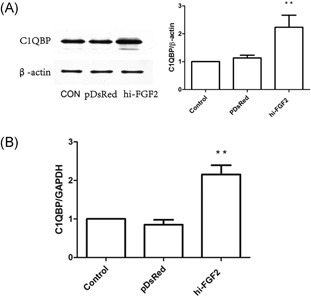
Hi‐FGF2 overexpression upregulates C1QBP expression. (A) C1QBP protein levels in hi‐FGF2–overexpressing and control HEK293 cells. Left, representative immunoblots showing C1QBP expression; β‐actin was used for normalization. Right, quantification of results. **P < .05 vs control cells (n = 3). B, *C1QBP* expression in hi‐FGF2–overexpressing and control HEK293 cells relative to *GAPDH* expression. **P < .05 vs control cells (n = 3). C1QBP, complement component 1 Q subcomponent–binding protein

We examined the effects of hi‐FGF2 overexpression on C1QBP expression to determine whether C1QBP is involved in hi‐FGF2–induced apoptosis. Western blot analysis showed a significant increase in C1QBP protein levels (*P* < .05) in hi‐FGF2–overexpressing cells compared with that of control and empty pDsRed‐transfected cells. Consistent with these results, quantitative reverse transcription‐PCR showed a significant increase in *C1QBP* messenger RNA expression (Figure 2).

### 3.3 Hi‐FGF2 interacts with C1QBP

In our previous study, we identified hi‐FGF2–associated peptides by carrying out affinity chromatography and mass spectrometry (MS)/MS peptide sequencing. As shown in Figure S2, proteins representing hi‐FGF2 (but not lo‐FGF2) interacting partners (indicated by arrows) were identified, 2 from the nuclear fraction and 8 from the cytosolic fraction (ranging from 21 to 45 kDa). The protein bands of interest were then excised and sent for high‐performance liquid chromatography‐MS for identification. One of the peptides identified by mass spectrometric analysis was the mitochondrial protein C1QBP. Mitochondria is involved in hi‐FGF2–induced chromatin compaction and cell death.^14^ Moreover, recent studies have shown that C1QBP modulates the opening of the MPT pore and thus mitochondria‐driven cell death.^17^, ^18^ Based on this information, C1QBP was selected for further analysis.

**Figure 3:**
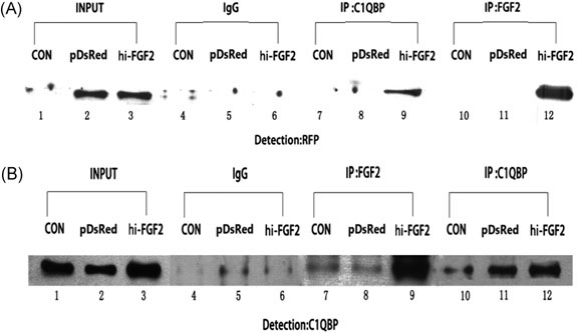
Hi‐FGF2 interacts with C1QBP in HEK293 cells. (A) CoIP of hi‐FGF2 and C1QBP. Western blot analysis was performed using anti‐C1QBP and anti‐RFP antibodies. CoIP was performed using 10% cell lysate as a loading control. Input control (lanes 1 to 3), rabbit IgG (lanes 4 to 6), and immunoprecipitated FGF2 (lanes 10 to 12). (B) CoIP of C1QBP and hi‐FGF2. Cell extracts were immunoprecipitated using the anti‐FGF2 antibody. Western blot analysis was performed using anti‐C1QBP and anti‐RFP antibodies. CoIP was performed using 10% cell lysate as a loading control. Input control (lanes 1 to 3), rabbit IgG (lanes 4 to 6), and immunoprecipitated C1QBP (lanes 10 to 12). CON, control; IgG, immunoglobulin G

HEK293 cell lysates were analyzed using anti‐C1QBP or anti‐FGF2 monoclonal antibodies. Proteins present in the cell lysates were resolved by SDS‐PAGE and then the expressions of RFP (Figure 3A) and C1QBP (Figure 3B) were analyzed by Western blot analysis. Next, we used C1QBP as the primary target to immunoprecipitate overexpressed hi‐FGF2 with the anti‐C1QBP antibody (Figure 3A; lane 9). Hi‐FGF2 did not coimmunoprecipitate with C1QBP in the control cells (Figure 3A; lane 7) or empty pDsRed‐transfected cells (Figure 3A; lane 8). Similar results were obtained using FGF2 as the antigen (Figure 3B). IP analysis using the anti‐C1QBP antibody produced weak signals with samples containing endogenous 23‐kDa FGF2 (Figure 3B; lanes 7, 8, 10, and 11). We conclude that C1QBP is able to interact specifically with hi‐FGF2.

### 3.4 C1QBP is crucial for localizing hi‐FGF2 to the mitochondria

**Figure 4:**
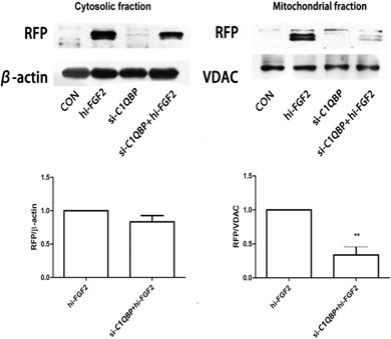
C1QBP is crucial for the mitochondrial localization of hi‐FGF2. VDAC (mitochondrial marker) and β‐actin (cytosolic marker) levels were used as controls. HEK293 cells were transiently transfected with si‐*C1QBP* for 3 days prior to Western blot analysis by using the indicated antibodies. CON, control nontransfected cells; C1QBP, complement component 1 Q subcomponent–binding protein; hi‐FGF2, hi‐FGF2–overexpressing cells; si‐*C1QBP*, *C1QBP* knockdown cells; si‐*C1QBP* + hi‐FGF2, cells transfected with si‐*C1QBP* and hi‐FGF2–expressing vector; VDAC, voltage dependent anion channel

Nuclear localization of hi‐FGF2 is required for chromatin compaction and induction of cell death.^14^ However, increased hi‐FGF2 levels have also been detected in the cytosol. C1QPB predominantly localizes to the mitochondria^16^, ^22^ but is also detected in the cytoplasm,^23^ on the cell surface,^16^, ^24^ and in the nucleus.^24^, ^25^ As C1QBP is usually a mitochondrial protein while hi‐FGF2 is predominantly localized to the nucleus, questions as for the subcellular compartment in which the 2 proteins may interact or colocalize arise. To address this, we fractionated cell lysates into cytosolic and mitochondrial fractions. Although mitochondrial fractions contain small amounts of cytoplasmic components, these components do not affect the results. With this fractionation method, we detected a subset of hi‐FGF2 protein in the mitochondrial fraction after the overexpression of hi‐FGF2 and high levels of C1QBP. Next, we knocked down *C1QBP* expression in HEK293 cells using specific siRNAs to determine the role of C1QBP in the mitochondrial localization of hi‐FGF2. *C1QBP* knockdown decreased endogenous C1QBP levels by approximately 70% to 80% but did not affect total FGF2 levels (data not shown). However, *C1QBP* knockdown significantly reduced mitochondrial hi‐FGF2 levels (Figure 4). Moreover, the rate of decrease in mitochondrial hi‐FGF2 levels was proportional to the decrease in *C1QBP* levels. These results show that hi‐FGF2 and C1QBP colocalize in the mitochondria and suggest that hi‐FGF2‐C1QBP interactions may be important for hi‐FGF2 mitochondrial localization.

### 3.5 C1QBP is a crucial mediator of hi‐FGF2–induced apoptosis

**Figure 5:**
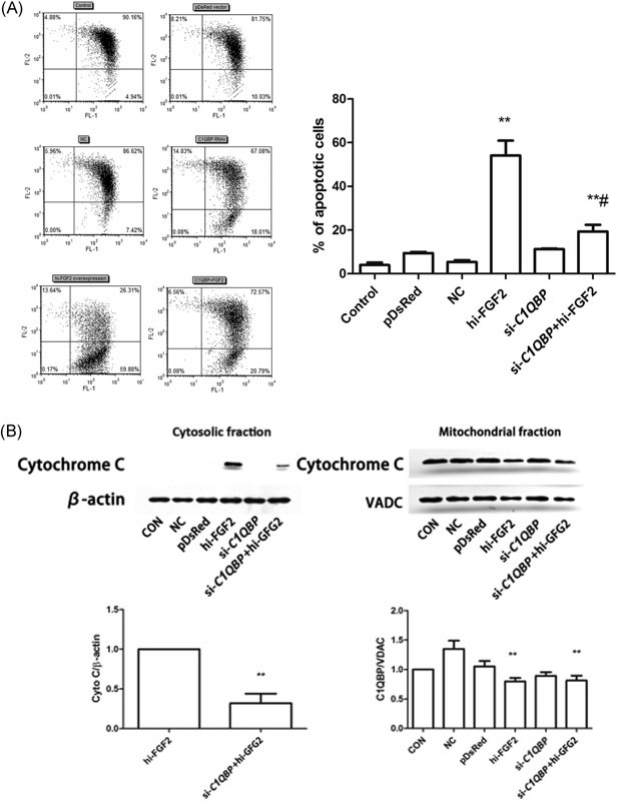
C1QBP expression is necessary for hi‐FGF2–induced apoptosis. (A) Flow cytometry analysis for determining mitochondrial membrane depolarization in HEK293 cells transfected with empty pDsRed vector, hi‐FGF2–expressing vector, si‐Scr–expressing, si‐*C1QBP*–expressing, or si‐*C1QBP* + hi‐FGF2–expressing vector. Right, percentage of apoptotic cells in different samples. Data are presented as the mean ± SEM (n = 3); **P < .001 vs control cells, ^#^ *P* < .01 vs hi‐FGF2–expressing cells. (B) Cytochrome *c* levels in cytosolic and mitochondrial fractions of previously mentioned samples. Data are presented as the mean ± SEM (n = 3); **P < .001 vs hi‐FGF2–expressing cells. Control, nontransfected cells; pDsRed, cells transfected with the empty vector; NC, cells transfected with si‐Scr; hi‐FGF2 overexpression, cells transfected with the hi‐FGF2–expressing vector; si‐*C1QBP*, cells transfected with si‐*C1QBP*; si‐*C1QBP* + hi‐FGF2, cells transfected with si‐*C1QBP*–expressing and hi‐FGF2–expressing vector; SEM, standard error of the mean

To elucidate the functional significance of the mitochondrial localization of hi‐FGF2 and its interaction with C1QBP, we examined the role of C1QBP in hi‐FGF2–induced apoptosis. We knocked down *C1QBP* expression using specific siRNAs before overexpressing hi‐FGF2 and then analyzed *C1QBP*‐knockout cells by flow cytometry. *C1QBP* knockdown significantly reduced mitochondrial depolarization (20.79%; *P* < .01; Figure 5A) compared with that of hi‐FGF2–overexpressing cells (59.88%; Figure 5A). Mitochondrial fractions isolated from hi‐FGF2–overexpressing HEK293 cells were analyzed for cytochrome *c* levels. We observed that *C1QBP* knockdown prevented the release of cytochrome *c* into the cytoplasm from the mitochondria (Figure 5B) compared with that of hi‐FGF2–overexpressing cells. Fractionation of mitochondrial and cytosolic proteins was confirmed by determining the VDAC and β‐actin levels as mitochondrial and cytosolic markers, respectively (Figure 5B).

## 4 DISCUSSION

A number of studies have reported a link between hi‐FGF2 overexpression in stable cell lines and cellular transformation.^5^, ^6^ On the other hand, several studies have suggested that this isoform is a potential inhibitor of cell proliferation^12^, ^13^ and inducer of cell death in certain cell types. Our results are consistent with those of a previous study that showed that increased hi‐FGF2 expression in NIH3T3 fibroblasts upregulated the expression of genes involved in cell‐cycle arrest, such as *NFLX* and *NUPR1*, and tumor suppression, such as *ST5*.^13^, ^14^ Moreover, hi‐FGF2 induces chromatin compaction and cell death by activating the mitochondria‐mediated proapoptotic pathway.^14^ As expected, based on previous studies, we found that nuclear hi‐FGF2 induces chromatin compaction, followed by overtly apoptotic‐looking nuclei after 48 hours in culture, Δψ_m_ depolarization, and cytochrome *c* release into the cytosol, indicating that hi‐FGF2 overexpression induced apoptosis. Additionally, increased TUNEL staining, engagement of mitochondria‐associated entities, such as the Bcl‐2 family of proteins, and accumulation of active capase‐3^14^ confirm that apoptosis was induced in HEK293 cells in response to hi‐FGF2 overexpression. Release of cytochrome *c* into the cytosol is possibly the result of Bcl‐2 protein family members (such as proapoptotic Bax or anti–apoptotic Bcl‐2) acting on the mitochondria. The effects of hi‐FGF2 may be attenuated in a background of Bcl‐2 overexpression, which would be expected to counteract the proapoptotic effects of Bax. In a similar manner, inhibition of Bax with a specific inhibiting peptide also attenuated the effects of hi‐FGF2 on chromatin compaction.^14^

Uncovering the protein‐protein interactions is important in understanding protein function. To further examine the specific effects of hi‐FGF2 on chromatin and cells in general, we identified proteins that interact with hi‐FGF2. Screening of GAL4‐based yeast 2‐hybrid expression libraries and CoIP assays identified FGF2‐interacting factor. The FGF2‐interacting factor–binding motif is located with the last 155 amino acids in the N‐terminal region of hi‐FGF2.^26^ Another study performed CoIP assays and immunofluorescence microscopy and showed that the survival of motor neuron protein interacts with the N‐terminus of hi‐FGF2 in Schwann cells and that hi‐FGF2 and survival of motor neuron protein colocalize in the nucleoplasm and nuclear gems.^19^ RG repeats in the N‐terminus of hi‐FGF2 are responsible for its nuclear localization^19^ and differential binding to different proteins. In the current study, we found that C1QBP levels were significantly increased in hi‐FGF2–overexpressing cells and interacted with vector‐expressed hi‐FGF2 but did not coprecipitate with endogenous hi‐FGF2. Our results indicate that the overexpression of exogenous hi‐FGF2 but not of endogenous hi‐FGF2 initiates a sequence of events, including the upregulation of C1QBP expression, which increases the mitochondrial localization of C1QBP and where exogenous hi‐FGF2 interacts with C1QBP. C1QBP was reported to be proapoptotic and is significantly upregulated during cisplatin‐induced apoptosis in HeLa cells.^20^, ^27^ Interactions between mitochondrial C1QBP and tumor suppressor p14/ARF^28^ or the prodeath Bcl‐2 family protein HRK^29^ are necessary for inducing mitochondria‐dependent cell death in cancer cells. Moreover, C1QBP can positively regulate UV‐induced apoptosis in HeLa cells and act as a positive regulator of mitochondrial calcium uptake in response to apoptotic stimuli, which was proposed to be mediated by a uniporter.^30^ In agreement with the proapoptotic role of C1QBP, C1QBP levels were found significantly increased in hi‐FGF2–overexpressing cells. Meanwhile, analysis of the mitochondrial fraction using the anti‐RFP antibody showed that the hi‐FGF2‐DsRed fusion protein localized to the mitochondria and that levels of this fusion protein significantly decreased in the mitochondrial fraction after *C1QBP* knockdown. This indicates that a subset of hi‐FGF2 putatively interacts with C1QBP via the N‐terminus and mobilizes it to the mitochondria, where C1QBP leads to decreases in the Δψ_m_.

Since C1QBP interacts with hi‐FGF2, we investigated the role of C1QBP in hi‐FGF2–induced apoptosis. *C1QBP* knockdown significantly decreased hi‐FGF2–induced apoptosis, as determined by measuring the Δψ_m_. Similarly, *C1QBP* knockdown increased cytosolic cytochrome *c* levels, indicating that a functional interaction between hi‐FGF2 and C1QBP is required for promoting hi‐FGF2–induced apoptosis. Numerous studies have shown that the ERK1/2 pathway is involved in apoptosis initiation, activation of the ERK1/2 pathway induces mitochondrial dysfunction during ceramide‐induced astrocyte apoptosis,^31^ and H_2_O_2_ treatment activates the ERK1/2 pathway in osteoblasts, further inducing mitochondrial apoptotic pathways.^32^

The ERK1/2 pathway is a major pathway stimulated by extracellular hi‐FGF2 and lo‐FGF2.^33^ The intracrine pathway of ERK1/2 activation is required for hi‐FGF2–triggered chromatin compaction and cell death in HEK293 cells.^14^ It is important to note that ERK activation may mediate an antiapoptotic function when activated by extracellular FGF2, as exogenous administration of lo‐FGF2 activates the ERK pathway^34^ and protects the heart from ischemia‐reperfusion–induced myocardial damage. It is possible that different pathways (intracrine versus autocrine or paracrine) can activate different pools of ERK1/2, affecting different downstream targets. However, the molecular mechanism by which intracrine hi‐FGF2 results in sustained activation of ERK1/2 is unknown. It is possible that hi‐FGF2 stimulates a pattern of gene expression that culminates in the upregulation of the ERK1/2 activation pathway. A study showed that intracrine hi‐FGF2 upregulated the expression of protein kinase C δ, which in turn activates ERK1/2.^35^ C1QBP is an endogenous substrate of mitogen‐activated protein kinase that translocates to the nucleus after PMA treatment in an ERK1/2‐dependent manner.^36^ Upregulation and nuclear translocation of C1QBP are essential for cisplatin‐induced apoptosis, which is suggested to be associated with ERK.^20^ Furthermore, C1QBP overexpression increases mitochondrial reactive oxygen species production, loss of Δψ_m_, cytochrome *c* release, and rat fibroblast death by opening the MPT pore.^37^ Thus, it is possible that the interaction of hi‐FGF2 with C1QBP induces mitochondria‐associated chromatin compaction and cell death through the ERK1/2‐dependent pathway.

**Figure 6:**
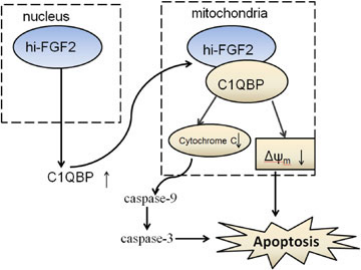
A schematic representing the mechanism of C1QBP in hi‐FGF2–induced apoptosis. A subset of hi‐FGF2 also localizes to the mitochondria through its N‐terminus and increases mitochondrial localization of C1QBP. Mitochondria‐localized hi‐FGF2 reduces Δψ_m_ and increases cytochrome *c* release, thus inducing apoptosis. C1QBP, complement component 1 Q subcomponent–binding protein

Taken together, our results reveal a novel mechanism by which cells overexpressing hi‐FGF2 can induce mitochondria‐associated apoptosis in HEK293 cells. This may be due to upregulated C1QBP expression and direct interactions on hi‐FGF2 with C1QBP, resulting in mitochondrial depolarization and subsequent cytochrome *c* release (Figure 6).

## Supporting information

Supporting information
