# Supplementary material for: High molecular weight fibroblast growth factor 2 induces apoptosis by interacting with complement component 1 Q subcomponent–binding protein in vitro
Source: J Cell Biochem. 2018 Aug 29;119(11):8807–17. doi: 10.1002/jcb.27131 (PMC6220755; doi:10.1002/jcb.27131)
Supplement: Supplementary file 1 — Supporting information [file JCB-119-8807-s001.docx]

Supplementary Information

**High molecular weight fibroblast growth factor 2 induces apoptosis by interacting with complement component 1 Q subcomponent-binding protein *in vitro***

Xiaobing Hong^1,2^, Zelin Yu^1^, Zhonglin Chen^2^, Hongyan Jiang^2^, Yongdong Niu^2^, Zhanqin Huang^2,^*

^1^*The Second Affiliated Hospital, Shantou University Medical College, Shantou 515041, Guangdong, China*

*^2^Department of Pharmacology, Shantou University Medical College, Shantou 515041, Guangdong, China*

*To whom correspondence should be addressed. E-mail: zqhuang@stu.edu.cn

**
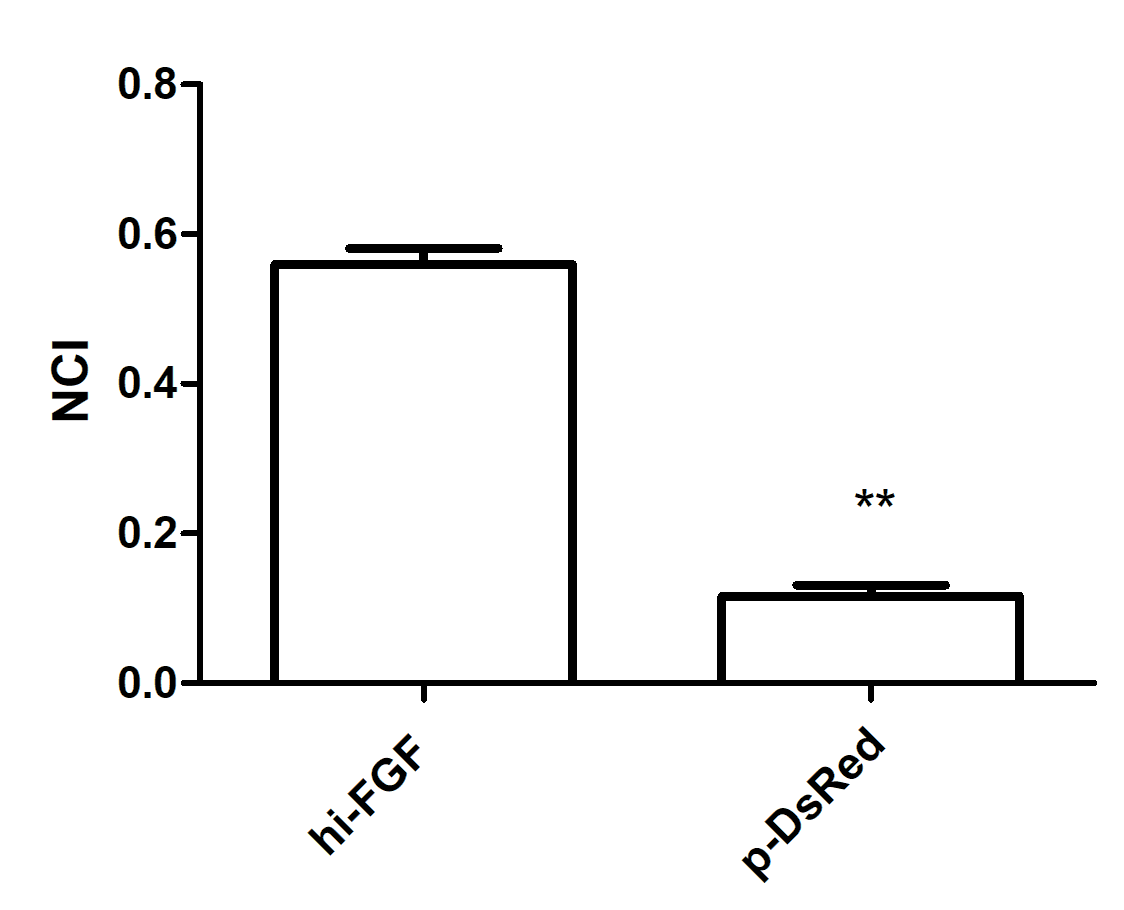
**

**Figure S1.** The nuclear compaction index (proportion of cells with compacted chromatin) is indicated for hi-FGF2–pDsRed1-N1-transfected cells and empty pDsRed1-N1-transfected cells at 48 h post-transfection. Data are presented as the mean ± SEM (n = 3); ***P* < 0.001.


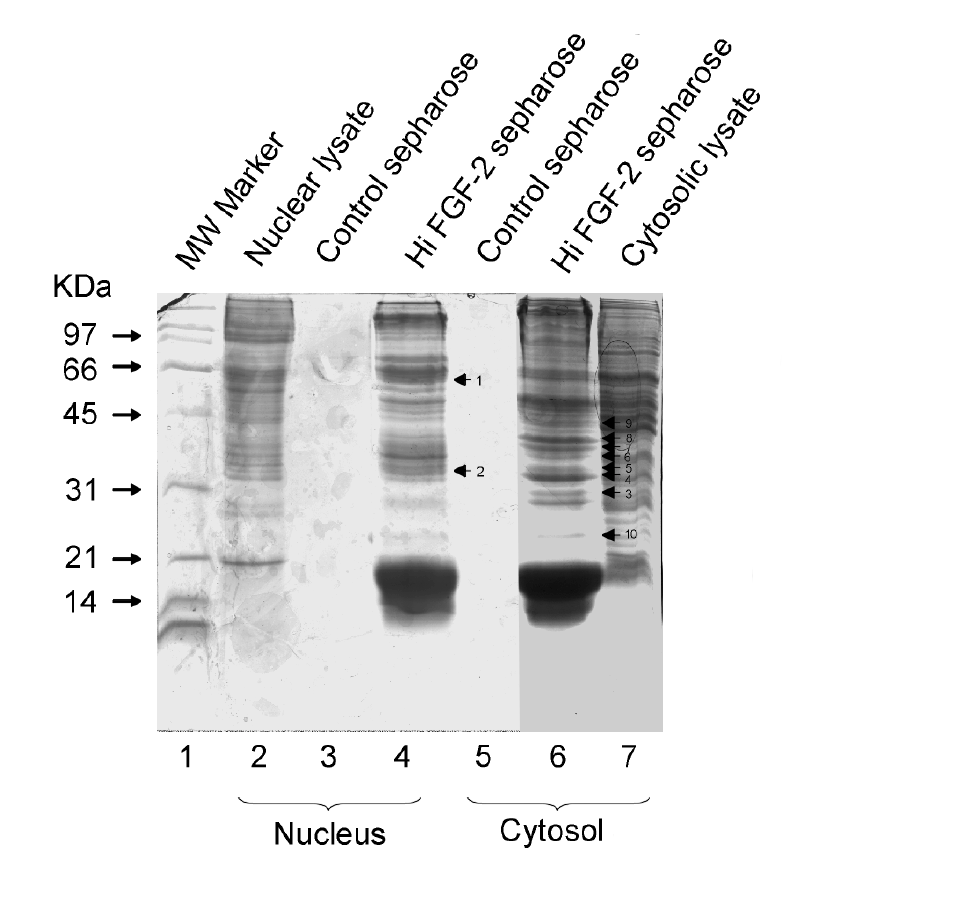


**Figure S2.** SDS/PAGE analysis of HEK293 nuclear and cytosolic proteins bound to hi-FGF2 affinity columns. Coomassie Brilliant Blue stained gel representing the following, lane 1, molecular weight markers; lane 2, total nuclear lysate, lanes 3 and 4, nuclear proteins bound to control-sepharose and hi-FGF2-sepharose, respectively; lanes 5，6, and 7, cytosolic proteins bound to control-sepharose, hi-FGF2 sepharose, and total cytosolic lysate, respectively. Arrows indicate proteins found in the hi-FGF2 sepharose-bound fraction and are labeled 1–7; they were excised and sent for identification by HPLC-mass spectrometry.
